# Recent Trends of Microfluidics in Food Science and Technology: Fabrications and Applications

**DOI:** 10.3390/foods11223727

**Published:** 2022-11-20

**Authors:** Ruojun Mu, Nitong Bu, Jie Pang, Lin Wang, Yue Zhang

**Affiliations:** 1 College of Food Science, Fujian Agriculture and Forestry University, Fuzhou 350002, China; 2 Key Laboratory of Subtropical Characteristic Fruits, Vegetables and Edible Fungi Processing (Co-Construction by Ministry and Province), Ministry of Agriculture and Rural Affairs, Shanghai 201106, China; 3 Department of Engineering Mechanics, Tsinghua University, Beijing 100084, China; 4 School of Food Science and Biotechnology, Zhejiang Gongshang University, Hangzhou 310018, China

**Keywords:** microfluidics, microencapsulation, spinning technology, functional food, emulsions

## Abstract

The development of novel materials with microstructures is now a trend in food science and technology. These microscale materials may be applied across all steps in food manufacturing, from raw materials to the final food products, as well as in the packaging, transport, and storage processes. Microfluidics is an advanced technology for controlling fluids in a microscale channel (1~100 μm), which integrates engineering, physics, chemistry, nanotechnology, etc. This technology allows unit operations to occur in devices that are closer in size to the expected structural elements. Therefore, microfluidics is considered a promising technology to develop micro/nanostructures for delivery purposes to improve the quality and safety of foods. This review concentrates on the recent developments of microfluidic systems and their novel applications in food science and technology, including microfibers/films via microfluidic spinning technology for food packaging, droplet microfluidics for food micro-/nanoemulsifications and encapsulations, etc.

## 1. Introduction

Food quality and safety are growing concerns in the food industry [1]. All steps during food processing are critical for achieving high-quality and safe food products [2,3]. Firstly, most food products are dispersed systems that contain various components. The development of food emulsions is one of the most efficient methods to enhance the quality of food systems [4,5]. In addition to traditional emulsions formed through homogenization, novel technologies have long been applied to produce emulsions in food systems, such as nanoemulsions produced through high-pressure microfluidization [6]. In food systems, there are also various minor components, such as polyphenols [7], probiotics [8], anthocyanins [9], etc., that are sensitive to stimuli during food processing. Microencapsulation is a common solution to increase the quality of food products [10,11]. Furthermore, food packaging is indispensable for processed whole food products [12,13]. It is a protection outside food systems and can effectively promote the product quality and safety of the food [14]. In addition, food analysis is the final step in processed food chains to ensure product quality and safety [15]. Various advanced methods have been applied in food analysis such as spectroscopic [8], electrochemical [16], and chromatographic methods [17]. However, all these techniques are relatively slow and require long times for sample preparation. The demand for cheap, high-throughput, and portable analytical systems has encouraged the development of new technologies and more suitable analytical methods.

**Figure 1:**
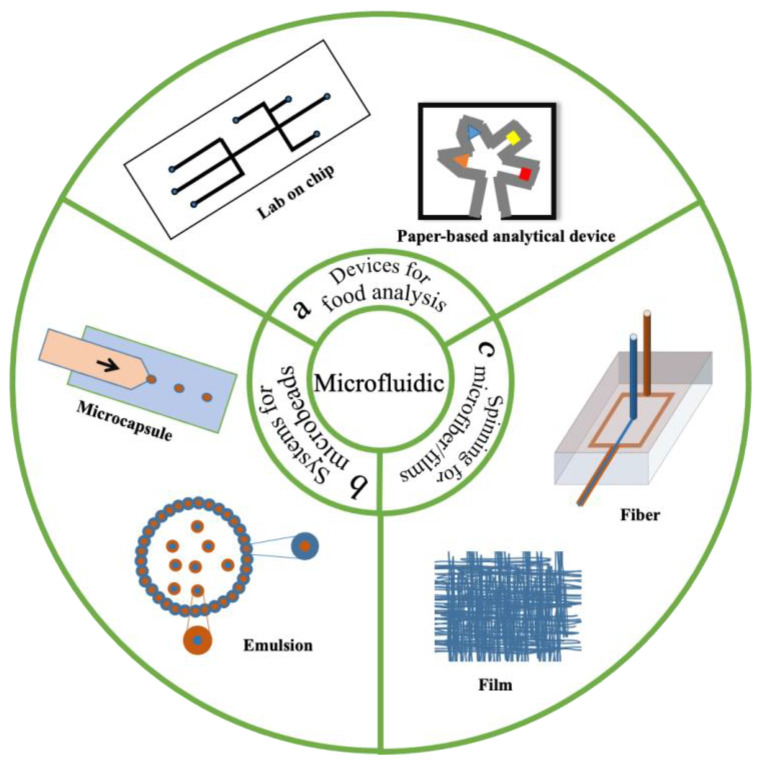
Potential applications of microfluidics in food science and technology. In this review, we conclude that there are three main applications of microfluidics: (**a**) microfluidic chips/devices for food analysis; (**b**) microfibers/films fabricated via MST; and (**c**) microcapsules and emulsions prepared from droplet microfluidic systems.

Microfluidics is an advanced technology for manipulating fluid flows in microscale channels (1~100 μm) [18]. A microfluidic system can be defined as a fluid element or chip in which there are many channels on the nanometer to micron scale [19]. These tiny channels give the fluid an interesting and unique character and a wide range of applications in different fields [20]. Microfluidic technology has shown great potential as a new tool in food science (Figure 1) [21,22]. Microfluidic technology can produce various shapes of micro–nano structures, including particles and fibers. Microfibers fabricated via microfluidic spinning have been used as microreactors for analyzing the chemical components [23], and microfilms have potential applications as functional packaging for food products [24]. Droplet-based microfluidics is an advanced technology to produce particles [25]. By controlling the wetting of the channel walls, this technology can generate stable emulsions that are widely applied in food systems [26]. In addition, droplet microfluidic strategy is used to manufacture advanced particles, which are useful structures for the encapsulation and release of active substances [27].

One previous review summarized the applications of microfluidic systems in food analysis [28]. Microfluidic technologies provide high-throughput and large-scale analysis via the integration of multiple steps, multiplexing, and the parallelization of analyses on a single device. Most analytical methods of components in food systems have been reviewed, such as major/minor nutrients, pathogens, toxins, and allergens, and thus are not included in this review. This review investigates the materials, design, and microchannel arrangement of microfluidic chips and their applications. This review focuses on the construction of microfluidic systems and their applications on micro-/nanofabrications in food quality assurance. The system is divided into two parts: The first one is microfluidic spinning technology, which can produce micro-/nanofibers and films. The second is droplet microfluidics, which can produce micro-/nanoemulsions and capsules.

## 2. Systems for Microfluidics

### 2.1. Microchannel Arrangements in Microfluidic Platforms

Microfluidics integrates basic operation units of sample fabrication, reaction, separation, and detection in the process of biological, chemical, and medical analysis into a microchip to automatically complete the whole analysis or preparation process [29]. The whole system for microfluidics is a combination of various unit devices that may include injectors (with a micropump), tubes and chips for the fluid flows, external stimuli systems for solidifications, and detection systems to collect signals from analysis [30]. The design of the microchannels of microfluidic chips is the main in many applications [31,32,33]. It is the key for achieving rapid analysis and multiple chemical reactions to produce various fibers and particles. The devices used for microfluidics have been developed using various materials. These devices exist in many forms, such as pulled-glass micropipettes, PDMS microchannels, metal needles, and tubes. Table 1 summarizes the platforms and microchannels applied (or potentially applied) in the fields of food processing and analysis.

**Figure 2:**
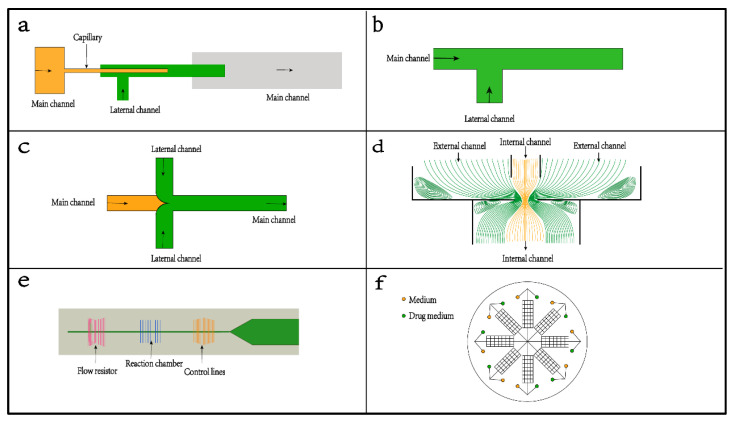
Microarrangements of platforms and devices in microfluidics: (**a**) a capillary-based platform that favors creating coaxial flows; three main arrangements in PDMS microfluidic chips: (**b**) T-junction, (**c**) cross-junction, and (**d**) concentric channel; and (**e**,**f**) two systems with a multichannel in a PDMS chip for analysis.

Microchannels are helpful to control the flow parameters in the process of coaxial flow [65]. Figure 2 illustrates various designs of microfluidic platforms. Capillary-based platforms (Figure 2a) are favored for creating coaxial flows for the production of microparticles and reactions in channels [66,67,68]. It is very simple; only two kinds of glass capillary tubes with different pipe diameters, glass sheets, syringes, and glue are needed to construct a capillary device. It should be noted that the axes of the two glass capillaries should be coaxial, as far as possible, to ensure a more stable and uniform liquid drop. In general, dispersed-phase fluid is introduced into the small-diameter capillary tube, and continuous-phase fluid is introduced outside of the small-diameter capillary tube. However, the fabrication of drawn glass micropipettes is labor-intensive and requires a high level of skill. In addition, those superthin tubes are easily cracked during the experiments [69]. The most developed microfluidic devices are PDMS channels or PDMS-based multifunctional platforms (Figure 2b–d). PDMS has high flexibility and chemical and thermal stability and is biocompatible with biological and medical applications [70,71]. The frequently used geometries are the T-connection (Figure 2b) and the cross-connection of four cross channels (Figure 2c). The T-connection uses the geometric structure at the intersection of the microchannel to make the front edge of the dispersed-phase fluid vertically enter the continuous-phase fluid. At the corner of the intersection, the momentum of the dispersed-phase fluid changes under the action of the continuous-phase fluid and finally becomes unstable. It is continuously sheared into many droplets [72]. Compared with the T-connection, the dispersed-phase in a cross-connection is subjected to continuous and symmetrical shear forces on both sides to produce a focusing effect and is extruded into droplets [73]. Compared with the T-connection, the cross-connections are more stable, convenient to operate, produce a wide range of droplet sizes, and are more likely to produce uniform small droplets much smaller than the channel’s characteristic size. The cross-connections are always filled with solutions and can solidify the polymers in the main channels. Both arrangements can produce microfibers [74,75,76] and microcapsules [77,78,79].

Two (or more) channels can be arranged concentrically to produce a strong extensional flow (Figure 2d). This arrangement is commonly applied for the formation of multiple emulsions and foams, which can be realized by one-step emulsification. Moreover, the stability of droplets can be improved. It has been proven that the coaxial channels have multifunctional advances: (1) Coaxial channels allow multiphase flows by confinement in microcapillaries and emulsification to multiple emulsion drops [80]. (2) A microfluidic platform with a coaxial annular chip interface is favored for the high-throughput production of emulsion droplets with controllable sizes and internal compositions [81]. (3) Coaxial channels can exert longer forces, allow larger deflections, and improve reliability [82]. In addition, a capillary-driven microfluidic chip with various and sophisticated arrangements is a common system for analysis or bioassay (Figure 2e,f). For example, Ezgi Ahi et al. fabricated a capillary-driven microfluidic chip with four continuous chambers for SERS-based hCG detection [83]. Li et al. prepared a stretch-driven microfluidic channel with multiple wing structures for nucleic acid detection [84].

### 2.2. Materials for Microfluidic Chips

The application of new materials and the combination and configuration of new technologies promote the development of microfluidic systems, which makes them more functional [85,86]. The materials for synthesizing microfluidic chips are mainly divided into the following categories: inorganic materials, organic materials, and composite materials (Table 2). 

Inorganic materials were first used as substrates in the early stage of microfluidics and usually had high surface stability, adjustable thermal conductivity, and solvent compatibility. Silicon and glass are the two most commonly used raw materials for chips. Silicon was the most ideal material due to its high possibility and advancement in micromachining technology [87,88,89]. However, silicon is an opaque material and has great limitations in optical detection [90,91]. In contrast, glass has strong light permeability with good surface chemical properties and high pressure resistance [92,93]. Various configurations of glass capillaries are often used in the microfluidic processing of functional microparticles [94]. Although glass materials have many advantages, the real challenge is how to use amorphous glass to prepare high specific surface area and anisotropic structural materials. Low-temperature cofired ceramic (LTCC) with laminar characteristic is another common inorganic material that can be utilized to prepare complex devices [95]. Currently, an LTCC device is usually applied in pharmaceutical analysis and sensors [96].

Organic materials are flexible with low costs. Compared to inorganic materials, they can make the process of microfluidic spin faster and simpler. The commonly used organic materials for microfluidic devices are polystyrene (PS), polyvinyl chloride (PVC), polymethyl methacrylate (PMMA), cycloolefin copolymers, polycarbonate (PC), and polydimethylsiloxane (PDMS) These materials have good surface modification, low thermal conductivity, and compatibility in biomedical applications [97,98]. PDMS microfluidic equipment has permeability to gas, and can therefore be used for long-term cell culture. According to their physicochemical properties, the organic materials used in microfluidic systems can be divided into the following categories: elastic [99], thermoplastic [100], plastic [101], hydrogel [102], and paper-based platforms [103]. While organic materials have many advantages, there are still some challenges in their applications, such as aging, chemical resistance, and their mechanical, optical, and thermal properties [101]. PDMS has high resistance to short-wavelength fluorescence detection, so the sensitivity of detection is much lower than that of glass materials [104].

The application of single materials such as silicon, glass, elastomers, and hydrogels can be combined into a hybrid chip to give full access to their advantages. The well-designed multifunctional complex system is suitable for different environments. Organic modified ceramics is a typical example that is beneficial to many applications of biological microfluidic technology [105]. Currently, the most widely used hybrid materials are based on the combination of PDMS with other materials, such as glass [106,107], SU-8 [108,109], polycarbonate [110], PMMA [111], hydrogel [112] and biodegradable materials [113,114,115]. Those hybrids can be applied in different states. A PDMS/polycarbonate microfluidic system uses a polycarbonate nanoporous membrane to control the fluid flow, which is also suitable for cell culture as a gas diffusion barrier [116]. Recently, scientists have developed an ethylene propylene polyimide film for the synthesis of organic materials. This film has high hardness under high pressures and good operability under low temperatures and is also chemically inert and will not react with most solvents [117].

**Table 2:** Summary of the common materials for microfluidic platforms.

Materials		Optical Clarity	Mechanics	Biocompatibility	Thermostability	Bonding Performance	Formability	Refs
Inorganic	Silicon	Good	Medium	Bad	Good	Difficult	Difficult	[118,119]
	Glass	Bad	Medium	Bad	Good	Difficult	Difficult	[120,121]
	LTCC	Bad	Medium	Good	Good	Difficult	Difficult	[95,96]
Organic	PDMS	Good	-	Good	Good	Easy	Easy	[122]
	PMMA	Good	Good	Good	Medium	Easy	Easy	[123,124]
	PC	Good	Good	Medium	Medium	Easy	Medium	[119,125]
	PS	Good	Medium	Bad	Medium	Easy	Easy	[118,119]
	PVC	Good	Good	Good	Medium	Easy	Medium	[118,119]
	SU-8	Medium	Good	Good	Good	Easy	Easy	[126,127]
Paper	-	Bad	Bad	Medium	Medium	-	-	[128,129]

## 3. Microfluidic Spinning Technology for Micro-/Nanofibers and Films

**Figure 3:**
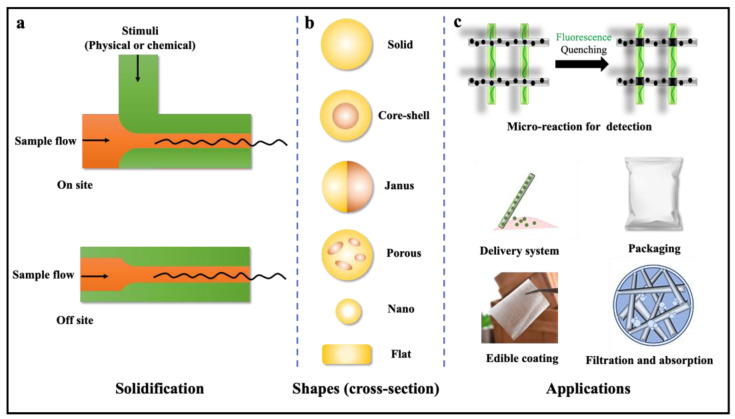
Fabrication of microfibers/films and their applications in food processing and analysis. (**a**) On-site and off-site solidification of microfibers. (**b**) Microfibers with diverse shapes. (**c**) Potential applications of microfibers for MST.

Microfluidic spinning technology (MST) refers to the preparation of microfibers with different sizes and morphologies from materials with a certain viscosity under the action of gravity by changing the fluid driving force and the drawing force of the receiver. MST has become a powerful and widely used platform due to its high specific surface area, effective heat transfer, and high reaction rate [130]. Microstructure fiber is very important because of its wide application, such as in microreactors, optical sensors, and biomaterials [23]. The multifunctional properties of microfluidic spun fiber show many potential applications in food science and technology. [131]. The advantages of microfluid devices for the fabrication of microfibers are as follows: (1) They are good systems to produce core–shell fibers or emulsion fibers to protect many sensitive components. (2) It is possible for the composites of different materials to enhance the mechanics of fibers and therefore form films for food packaging. (3) To make the best use of microfilms, MST can introduce bioactive molecules into the fibers to improve their functionality (such as antioxidation and antimicrobial properties). (4) Functional microfibers/films produced from MST are also good systems for food analysis and solvent purification. Figure 3 and Table 3 briefly illustrate the fabrication of microfibers via microfluidic spinning technology and its potential applications in food processing and analysis.

### 3.1. Solidification Methods for Microfiber Generation

Solidification is critical for the formation of microfibers. The diversity of approaches to solidify polymer solutions to generate functional microfibers/films has been investigated for a long time. Two reviews simply divided the methods of solidification into photopolymerization and chemical reactions [132]. Photopolymerization mainly uses UV light to crosslink prepolymers such as poly (ethylene glycol), diacrylate (PEG-DA), and 4-hydroxybutyl acrylate (4-HBA) then generate the solidified microfibers. Chemical reactions always introduce a crosslinking agent to react with prepolymers and form solid fibers. For example, sodium alginate is crosslinked due to the diffusion of calcium ions into a sodium alginate solution. In their review, the solidification approaches were divided into chemical reactions and physical processes. In this classification, photopolymerization was collected into the chemical reactions, as chemical reactions happen during the polymerizations. The physical processes include ionic crosslinking, solvent exchange, non-solvent-induced phase separation, and solvent evaporation. Based on the solidification time during the fabrication of the microfibers, we divided them into two groups (Figure 3a). The on-site solidification shows that the microfibers are solidified inside the devices. A physical or chemical stimulus (such as UV light, ions, reactive agents, etc.) is applied during the fabrication of the microfibers. The off-site solidification does not apply any physical or chemical approach inside the devices. This fabrication approach always needs a solid receiver for the microfibers because they can only be solidified after solvent evaporation. Many food materials have been used for the solidification of microfibers, such as alginate, chitosan, glucomannan, and silk proteins. Those fibers with various shapes and functions were applied in different areas of science and engineering.

### 3.2. Design Principle of Fibers with Different Shapes

MST is distinguished by constructing micro-/nanofibers with complex shapes (solid, core shell, Janus, hollow, nano, flat, etc.) by simply regulating the channel shape, solvent type, solidification methods, flow rates, types, concentration of spinning solution, etc. [133].

It is convenient to prepare solid-shaped fibers through a single channel and a coaxial multichannel. The size of fibers is commonly influenced by the dimensions of channels, the flow rates of spinning solutions, and the solidification methods. In general, the size of a fiber mostly depends on the size of the spinning fluid in the channel in the range of micrometers to one hundred micrometers [130]. When using different solidification methods, such as on-site photopolymerization, it can also effectively adjust the size of fibers by altering the intensity of irradiation and irradiation time [134]. The on-site ionic crosslinking-assisted fabrication of biological fibers presents potential advantages in the fast gelation process. The Ca^2+^ cross-linker is one of the most commonly applied agents for this gelation process. It has been used for the solidification of mixed carbon nanotubes and sodium alginate to fabricate nanofiber-based macroscopic cables [135]. In addition, the size of fibers was also influenced by the types of solvents and the concentrations of spinning solutions [136]. One recent study prepared silk fibers with sizes ranging from 54.1 to 102.2 nm by non-solvent-induced phase separation [137].

It is necessary to add other capillary tubes for the injection of different fluids to generate core–shell and hollow fibers. In a previous report, researchers constructed hollow fibers utilizing three microchannels, where the innermost tube was filled with a CaCl_2_ solution. The second inner tube contained a sodium alginate solution, and the outermost flowed with a CaCl_2_ solution. The hollow fiber was formed by two stages of gelation at the intersection of the tubes, where the core sodium alginate solution gelled with the CaCl_2_ solution in the innermost tube, followed by gelation with the CaCl_2_ solution in the outermost tube [138]. Based on hollow fibers, core–shell fibers can also be prepared by MST by combining a curable core fluid and a polymerizable shell fluid. For instance, Daniele et al. produced UV-assisted core–shell fibers composed of polyethylene glycol dimethacrylate (shell) and gelatin (core) [139]. To further precisely manipulate the thickness of each layer of the core–shell fibers, the multiphase flow rates should be strictly controlled. Researchers found that decreasing the concentration of gelatin could lead to a shape change for the fibers. Janus fibers can be generated by multiphase microfluidic platforms composed of outer sheath flow inlets and sample flow inlets. One common approach for the preparation of Janus fibers is to inject a photocurable PU solution into the central microchannel and simultaneously inject a sodium dodecyl sulfate solution into the outer sheath microchannels. Hence, Janus fibers were formed under UV irradiation [140]. Flat-shaped fibers can also easily be produced by MST. Lee and coworkers investigated whether MST can fabricate microbelt-shaped fibers consisting of poly(ethylene glycol) diacrylate. During the processing, several poly(ethylene glycol) diacrylate streams were simultaneously introduced into inlets and generated multiphase jetting without the engulfment phenomenon due to their similar compositions [141]. 

### 3.3. Applications of Microfibers in Analysis and Encapsulations

Microfibers with diverse shapes (such as cylindrical, flat, grooved, porous, core–shell, etc.) (Figure 3b) fabricated via MST perform many functions (Table 3). The most easily prepared cylindrical microfibers allow chemical reactions to be performed inside to form microreactors or sensors for analysis. Yu et al. described a high-throughput photofluidic platform of mosaic patterned microfibers by generating a layered laminar flow and prepared mosaic microfibers with the desired configuration for multiple biomolecular analyses [142]. Mu et al. proposed an MST for the fabrication of microfibers with KGM and sodium polyacrylate (PAAS) [23]. 

The prepared microfibers can be easily arranged into microarrays and microgrids, which provides a useful platform for amine molecular recognition. The function of microfluidic fibers also depends on the inclusion of functional components. The encapsulation of functional components is one of the commonly used methods for the functionalization of microfibers, which can further expand the application prospect of microfibers. In general, functional components are added to a curable precursor solution to obtain a uniform suspension or a uniform solution as a sample stream. Then, the fibers with expected properties are obtained by MST [130]. By encapsulating various functional components, those microfibers obtain unique properties that are applied in tissue engineering and the biomedical area. It has been well-investigated that the microfibers produced via MST are good delivery systems for cells. One study successfully prepared chitosan–alginate fibers carrying human liver cancer cells (HepG2) using a coaxial flow microfluidic chip [143]. Another recent work described a new alginate microfiber production method using microfluidic technology to precisely regulate microfibers, thereby improving the vitality and function of embedded cells [144]. In order to protect the transplanted islets immunologically, an immunoprotected ultrafine fiber has been newly designed for successful islet transplantation [145]. The most commonly used alginate-Ca^2+^ solidification produced many functional microfibers that can potentially be applied in food science. For example, Chaurasia and Sajjadi made alginate fibers loaded with oily objects through the arranged internal capillaries, showing the potential for better triggering reactions [146]. Meng et al. reported an effective microfluidic method for the successive production of hollow calcium alginate microfibers with controlled structure and function, and the addition of active components to the core stream for encapsulation [147]. Pham et al. proposed a simple method for the self-assembly of hollow alginate microfibers based on a PDMS microfluidic device [148]. The inner diameter and wall thickness of the microfibers were controlled by changing the flow velocity of the core and sheath in the microfluidic channel.

**Table 3:** Summary of the applications of micro-/nanofibers and films in food processing and analysis.

Materials	Solidification	Shape	Functional Component	Applications	Refs
Konjac glucomannan/polyvinylidene fluoride	Off-site	Solid	Epigallocatechin-3-gallate	Drug release	[63]
Konjac glucomannan/sodium polyacrylate	Off-site	Solid	Ofloxacin	Microreactors	[23]
Konjac glucomannan/polylactic acid	Off-site	Solid	Trans-cinnamic	Food packaging	[24]
Konjac glucomannan/poly(ε-caprolactone)	Off-site	Solid	Silver nanoparticles	Food packaging	[149]
Konjac glucomannan/poly (methyl methacrylate)	Off-site	Solid	Chlorogenic acid	Food packaging	[150]
Ethyl cellulose/polyvinylpyrrolidone	Off-site	Solid	-	Food packaging	[64]
Polyurethane/sodium dodecyl sulfate	On-site	Janus	-	3D scaffold	[83]
Graphene oxide/ bacterial cellulose	On-site	Core–shell	Silver nanoparticles	Antibacterial	[151]

### 3.4. Applications of Microfilms as Food Packaging and Purification Systems

Microfibers can be further integrated to form functional films and exhibit various properties such as high mechanical, antioxidation, and antimicrobial properties. These microfilms have potential applications in food packaging [152]. In our group, we prepared microfilms based on a new kind of biopolymer, KGM, which also promotes wound healing through new blood vessel formation and the advanced development of hair follicles [153]. We further adopted simple and green strategies and built a series of triple-layer microfilms, such as a konjac glucomannan/polylactic acid/anti-cinnamic acid micromembrane (KPTMF) [24] and a konjac glucomannan/poly (methyl methacrylate)/chlorogenic acid food packing film inspired by the amphiphilic theory [150]. In addition to the packaging, microfilms produced via MST are also applied in purifications. Inspired by the conformation of spider silk, He et al. manufactured ultrafine fibers with adjustable magnetic spindle-knotted microfibers [154]. Those fibers contained magnetic Fe_3_O_4_ nanoparticles for controlled 3D assembly and water collection. Wu et al. utilized microfluidic emulsification and spinning synergy technology to prepare spindle-knotted graphene microfibers that can absorb oil in the environment of a water–oil mixture [155]. Graphene oxide (GO) fibers with a spindle junction structure and photothermal response phase change behavior are manufactured in microfluidics through spinning and emulsification programs, which can realize fog capture and near-infrared-triggered water collection applications [156].

## 4. Droplet Microfluidics for Micro-/Nanoemulsions and Capsules

**Figure 4:**
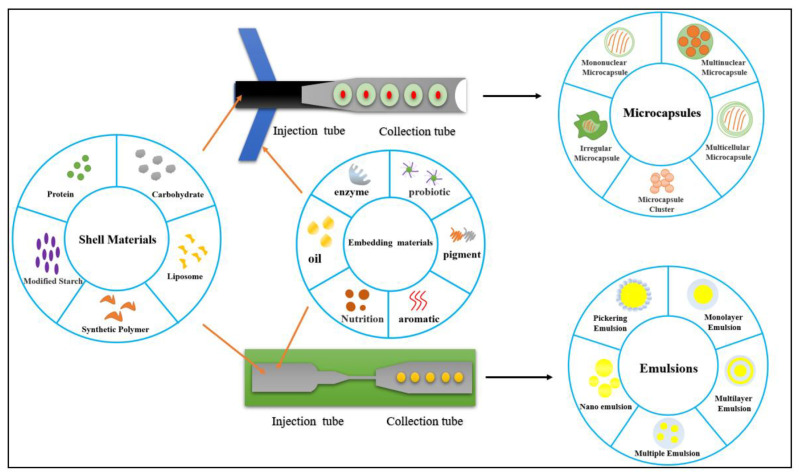
Droplet microfluidics for emulsions and microcapsules.

The continuous production and processing of monodisperse microparticles have always been a scientific and technical issue. The first advantage of microfluidic equipment in the fabrication of food products is that it can provide a suitable processing environment for fluids. In microchannels, food scientists can obtain stable microparticles in a homogeneous emulsion by controlling the fluid to give them corresponding physical and chemical properties [157,158]. Therefore, microfluidics has become a widely used advanced technology for the preparation of microparticles. Another advantage is that this technology can not only prepare single-component gel particles but can also prepare mixed-component particles with specific structures and characteristics. In materials science, droplet microfluidics has been applied to fabricate “Janus” microbeads [159], core–shell microcapsules [42], porous microparticles [160], and photosensitive [161] and thermosensitive [162] capsule particles. The third advantage is that the stop and flow technology of a microfluidic system in a flat plate is creating a revolutionary change in the field of material processing [163]. Traditional material processing technology can only synthesize spherical materials because of the effect of surface tension in the emulsion system. In microfluidics, fluid can be projected into different patterns in the process of microchannel chip flow [164]. This technology has been applied to the preparation of many microparticles with various shapes, including cell-loaded microparticles, some column-shaped evolution particles, high-throughput particle processing, and combination with fluid aggregation or a large-scale array [165]. Additionally, the use of a microfluidic nebulizer can achieve the application of ultrasonic spray-drying technology, synthesized RNA-loaded lipid nanoparticles, and microreactors for controlling the physical and chemical changes in the material synthesis process [166]. Figure 4 and Table 4 illustrate droplet microfluidics for emulsions and microcapsules.

### 4.1. Principle of Droplet Microfluidics

The fluid behavior in droplet microfluidics is mainly divided into laminar flow and droplets [167]. Hence, it is necessary to introduce a driving force to push the fluid into a microchannel. Pressure is the one of the common driving forces urging the flow at the import and export of the channel. We can simply adjust the parameters of the syringe pump to control the driving forces applied to the fluid. Apart from external driving forces, the flow of fluid can be promoted by its gravity [168]. When introducing multiphase fluids into a microchannel, a droplet is generated by the energy from the breakup of fluids, which provides interfacial energy for the production of emulsions [169]. In this process, there are two types of methods: passive methods (e.g., coflow, cross-flow, and flow-focusing) and active methods (e.g., electrical, magnetic, thermal, and mechanical methods), which are mainly distinguished by the existence of external energy. The principle of droplet microfluidics was reviewed in detail in a previous report [26].

Microcapsules and emulsions generated by droplet microfluidics have been widely applied in the field of encapsulation and release. In order to construct microcapsules and emulsions, a T-junction or cross-junction is usually needed, where the core materials flowing into the main tube are wrapped by the core materials flowing into lateral tubes. It is feasible to regulate the size and number of inner droplets by controlling the flow rates. Apart from the assembly of droplets, designing the multistructure of droplet microfluidic devices is another effective strategy. A capillary device combined with continuous narrowing expansion junctions is an applicable example. In brief, oil droplets are first produced by flow-focusing. Then, oil droplets flow across continuous narrowing expansion junctions, where the spherical oil droplets are first squeezed at the narrow export, allowing the inclusion of water, then relaxed into a spherical shape at the expansive import, and eventually formed into double emulsions [170]. Another strategy is to combine “co-flow” and “flow-focusing into” in one capillary device, where two immiscible fluids flow coaxially and another outer immiscible fluid flows in the opposite direction [171]. When three fluids flow simultaneously through a tapered tube, double emulsions are formed.

### 4.2. Droplet Microfluidics for Microcapsules

Microparticles are becoming increasingly important tools for a wide range of applications in food science, such as probiotic and nutrient delivery [78,172]. Microencapsulation is an important technology for sustaining cell viability and nutrient activity during food processing [10,11]. Microencapsulation fixes the surface of the emulsion to enhance the stability of the emulsion and cover the solid, liquid, or gas in a shell to protect or isolate the material in the shell, prevent the external environment from producing adverse or toxic effects on the material in the shell, and control the release of the material in the shell [173]. The core of a microcapsule is generally the cavity or the active substance dispersed in the cavity, which can be one or multiple substances. The shells may be single, double, or multiple layers and are usually made of organic polymers [174]. The capsule shell can not only protect the active ingredient from the damage of pH change, oxidation, water, and other environmental pressures but also regulates the release of the active ingredient. The microcapsule shape is relatively rich, including spherical, a grain shape, and amorphous, but a spherical structure is the most common structure. Microcapsules can be divided into mononuclear microcapsules, multicompartment microcapsules, irregular microcapsules, etc. 

The development of a new type of biomaterial is an urgent need for the application of food science. Especially in the field of food science, using low-cost and biocompatible raw materials is a challenging task [175]. Polysaccharides are a kind of robust biopolymer extracted from natural sources. Several of them are ideal materials for application in microfluidic technology, such as sodium alginate [78], chitosan [176], and konjac glucomannan [24]. Among various possibilities, alginate gel particles are widely used. They are nontoxic, biocompatible, biodegradable, inexpensive, and relatively simple to produce, especially as carriers of microencapsulated compounds [177,178,179].

### 4.3. Droplet Microfluidics for Emulsions

Food emulsions exist in most food systems [180,181,182]. The easiest double emulsion system from microfluidics has long been applied to stabilize the sensitive compounds in food. For example, Xu and Nisisako fabricated double emulsions using a microcapillary device containing a single internal droplet in a core–shell geometry [183]. Nam et al. utilized poly(ethylene glycol) diacrylate (PEGDA) and droplet microfluidics to obtain the direct encapsulation of water and oil droplets in PEG microcapsules [184]. Recently, one study compared the emulsion systems between microfluidics and the conventional homogenization method. The microcapsules obtained via microfluidics exhibited many advances, such as monodispersity, the stability of physical and chemical properties, and the tracking ability of shell crosslinking [44]. To obtain more stability and various functions of the emulsions, multiple layers of emulsions were developed in food systems. For example, Kantak et al. described a new microfluidic technology that utilizes a microcolumn in the channel to continuously generate, encapsulate, and guide layer-by-layer (LBL) polyelectrolyte microcapsules [185]. In less than 3 min, six hydrogen-bonded polyelectrolytes (three double layers) were adsorbed on each droplet. A new technology recently introduced an ultrathin water layer between two phases in a triple emulsion and achieved high packaging efficiency of hydrophobic goods in a hydrophobic polymer shell directly dispersed in water [186].

**Table 4:** Summary of the droplet microfluidics for emulsions and microcapsules in food processing and analysis.

Shell Materials	Embedding Materials	Type	Applications	Refs
Polycaprolactone	Chlorophyll	Microparticle	Drug encapsulation	[47]
Starch	Nisin	Nanoparticle	Drug encapsulation	[48]
Silk fibroin/chondroitin sulfate/alginate	Bovine serum albumin/polystyrene latex	Microgel	Drug delivery	[51]
4,4-methylenediphenyl diisocyanate/ethylenediamine	Pendimethalin	Microcapsule	Drug delivery	[187]
Chitosan	Curcumin/catechin	Microcapsule	Drug delivery	[188]
Sodium alginate	Sucralfate	Microcapsule	Intestinal barriers	[189]
Zein	Nisin	Microcapsule	Drug encapsulation	[190]
Zein	Lecithin	Microcapsule	Drug delivery	[191]
N-isopropylacrylamide/methacrylic acid	Lumogen Red	Microcapsule	Drug delivery	[192]
Liposomes	Plasminogen activator	Liposome	Drug encapsulation	[193]
Sodium alginate/gelatin	Vitamin A	O/W emulsion	Drug encapsulation	[194]
Sodium alginate/cellulose nanocrystals	Oil	O/W emulsion	Lipophilic compound delivery	[195]
Gelatin	β-carotene	O/W emulsion	Drug encapsulation	[196]
Polyvinyl alcohol	Rifampicin	W/O/W emulsion	Drug delivery	[27]
PDMS-*b*-PDMAEMA	Sucrose/catechin	W/O/W emulsion	Drug encapsulation	[34]
Sodium alginate	Phycocyanin	W/O/W emulsion	Drug delivery	[42]
Sodium alginate/calcium–ethylenediaminetetraacetic acid	Oil	O/W/O emulsion	Lipophilic compound delivery	[197]
Silica nanoparticles/poly(diallyldimethylammoniumchloride)/polystyrene sodium sulfate	Trypsin	W/W emulsion	Enzyme delivery	[198]

## 5. Conclusions and Perspectives

Microfluidics has been defined for more than 60 years. Due to the unique fluid properties in microscale environments, this technology is widely applied in food processing. Microfluidics can produce fine droplets to stabilize the multicomponents of dispersed systems, which is very beneficial for the design of novel emulsion-based foods. Compared to the traditional phase-dispersion processing, microfluidics is more effective in the use of energy as well as the control of the shape, size, and size distribution of components. It has the potential to significantly change the processing of dispersed food systems. In addition to emulsions, microfluidics is also an efficient approach to prepare solidified microcapsules for protecting sensitive objects during food processing and storage. Microfibers/films fabricated by microfluidic spinning technology with high mechanical properties can potentially be applied as functional packaging for various food products. The bioactive components dispersed or embedded in the fibers/films are critical factors for food preservation and analysis. On-chip microfluidic systems also provide high-throughput and large-scale analysis by integrating multiple steps, multiplexing, and parallel analysis in a single device.

It is obvious that the microfluidics can be applied in many fields of food science. However, most current applications of this new technology are in the development of functional emulsions. Fibers and films are still in a lab-scale stage, and there are still many attempts expected from food scientists to scale-up and expand its influences in food science: (1) Microcapsules produced via microfluidics have been applied for embedding many sensitive components in food systems. However, those applications are used far less than traditional methods. More components can be stabilized by introducing this easy-to-use, stable, and cost-effective technology to produce functional foods. (2) Microfluidic spinning technology is an efficient way to fabricate microfibers/films. Their potential applications as functional packaging and microanalysis systems have already been demonstrated by recent studies from our groups and other peers. However, the solidification of biopolymers (such as proteins and polysaccharides) and their stabilities during the processing and application of micro-/nanofibers is still a challenge. More investigations need to be focused on the design of biopolymers to produce stable fibers and films. (3) There is no doubt that both MST and droplet microfluidics have aroused wide attention in the field of food science and technology. However, most applications are currently in the laboratory stage of development. One challenge for large-scale production in industry is the design of the microfluidic equipment. In a lab, a chip can be easily produced for the fabrication of microfibers and droplets. One cannot guarantee that the same design of microchannels will be applicable in industry. Another challenge is productivity of the microfluidic equipment. Demonstration is always needed in fundamental research, but mass production is needed in industry. Although some strategies can fulfill the requirement of ultrahigh throughput, much more effort should be devoted to exploring the manipulation of the fiber and droplet generation under complex flows in an extended scale of microchannels in industry.

## Figures and Tables

**Table 1:** Summary of the microfluidic platforms applied in food processing and analysis.

Material	Type (Microchannel Arrangement)	Applications	Refs
PDMS	Cross-junction	Construction of emulsions and encapsulation of active substances	[34]
	Y-junction	Controlled assembly of nanoparticles and drug delivery	[35]
	Multichannel	Screening of drugs	[36]
	Multichannel	Detection of pathogens	[37]
	Multichannel	Immunoassay	[38]
Glass	Capillary	Creation of food assemblies	[39]
	Capillary	3D bioprinting	[40]
	Capillary	Construction of multiple emulsions and protection of natural pigments	[41]
	Capillary	Encapsulation, protection, and delivery of protein	[42]
	Capillary	Construction of nanoparticles for pharmaceutical delivery	[43]
	Capillary	Preservation of oil	[44]
	Capillary	Encapsulation of food ingredients	[45]
	Cross-junction	Content analysis of protein	[46]
	Cross-junction	Enhancement of the stability of pigment	[47]
	T-junction	Encapsulation of polypeptide	[48]
Glass-based hybrids	Capillary	Detection of pathogens	[49]
	Cross-junction	Generation of emulsion	[50]
	Cross-junction	Delivery of nanocarriers	[51]
	Serpentine channel	Detection of protein	[52]
Paper	Multichannel	Monitoring systems of food adulterants	[53]
	Multichannel	Detection of drugs	[54]
	Humped circle	Detection of pathogens	[55]
	Syringe needle	Delivery of drugs	[56]
	Cross-junction	Water-in-oil emulsification	[57]
PMMA	Multichannel	Extraction of DNA	[58]
Polycarbonate	Capillary	Extraction of DNA	[59]
Fused silica	Serpentine channel	Detection of nucleic acids	[60]
	Hollow microneedle	Delivery of drugs	[61]
Stainless steel	Syringe needle	Wound dressing	[62]
	Syringe needle	Release of drugs	[63]
	Syringe needle	Microreactor for amine detection	[23]
	Syringe needle	Food packaging	[64]

## Data Availability

Not applicable.
